# Predicting Decisions in Human Social Interactions Using Real-Time fMRI and Pattern Classification

**DOI:** 10.1371/journal.pone.0025304

**Published:** 2011-10-07

**Authors:** Maurice Hollmann, Jochem W. Rieger, Sebastian Baecke, Ralf Lützkendorf, Charles Müller, Daniela Adolf, Johannes Bernarding

**Affiliations:** 1 Medical Faculty, Institute for Biometry and Medical Computer Science, University Magdeburg, Magdeburg, Germany; 2 Department of Neurology, Medical Faculty, University Magdeburg, Magdeburg, Germany; 3 Department of Neurology, Max Planck Institute for Human Cognitive and Brain Sciences, Leipzig, Germany; Institute Biomedical Research August Pi Sunyer (IDIBAPS)-Hospital Clinic of Barcelona, Spain

## Abstract

Negotiation and trade typically require a mutual interaction while simultaneously resting in uncertainty which decision the partner ultimately will make at the end of the process. Assessing already during the negotiation in which direction one's counterpart tends would provide a tremendous advantage. Recently, neuroimaging techniques combined with multivariate pattern classification of the acquired data have made it possible to discriminate subjective states of mind on the basis of their neuronal activation signature. However, to enable an online-assessment of the participant's mind state both approaches need to be extended to a real-time technique. By combining real-time functional magnetic resonance imaging (fMRI) and online pattern classification techniques, we show that it is possible to predict human behavior during social interaction *before* the interacting partner communicates a specific decision. Average accuracy reached approximately 70% when we predicted online the decisions of volunteers playing the ultimatum game, a well-known paradigm in economic game theory. Our results demonstrate the successful online analysis of complex emotional and cognitive states using real-time fMRI, which will enable a major breakthrough for social fMRI by providing information about mental states of partners already during the mutual interaction. Interestingly, an additional whole brain classification across subjects confirmed the online results: anterior insula, ventral striatum, and lateral orbitofrontal cortex, known to act in emotional self-regulation and reward processing for adjustment of behavior, appeared to be strong determinants of later overt behavior in the ultimatum game. Using whole brain classification we were also able to discriminate between brain processes related to subjective emotional and motivational states and brain processes related to the evaluation of objective financial incentives.

## Introduction

Neuroscientific studies of the brain mechanisms of social decision-making offer new insight which helps to incorporate human behavior into economic models. In the framework of neuroeconomics, cognitive and neural constraints of the complex processes of social decision-making are explored [1]–[5]. Experimental paradigms from game theory are well suited to the investigation of neural correlates of decision-making, because profound empirical insight into human behavior is provided [1], [6].

Using a real-time noninvasive technique based on fMRI, we investigated the neural correlates of social decision-making and tried to already infer the decisions made by participants involved in social interaction from brain activation during scanning. We employed a well-established economic game called the ultimatum game (UG), in which two players split a given amount of money. One player acts as the proposer, retaining one share of the money and offering the remaining share to the other player (the responder). The responder can either accept or reject the proposer's offer. If the offer is accepted, the money is split as proposed. If the offer is rejected, neither player receives anything. According to the notion of profit maximization, the proposer is expected to offer the smallest possible sum of money and the responder to accept this offer, because even the smallest profit is preferable to no monetary reward [6]. Contrary to this assumption, it has been repeatedly shown that the results of negotiation in this game do not conform to the expected game-theoretic equilibrium outcomes. Instead, low (unfair) offers of 10–20% of the total sum of money are rejected in more than 50% of cases [6], [7], suggesting that emotions, attitudes, and expectations influence players' decisions.

Social interaction as in the ultimatum game may lead to conflicts between players' goals and internal attitudes and social norms, which elicit emotions. These conflicts require considerable cognitive effort to be resolved [2], [8], [9]. Consequently, previous fMRI studies on decision-making report the involvement of cortical and subcortical brain regions related to cognitive control, such as prefrontal cortex, anterior cingulate cortex, and regions connected to emotional response such as amygdala and insular cortex (for a review see [1]). Decision-making processes in social interaction scenarios have already been examined using functional magnetic resonance imaging (fMRI) [3], [4], [10], [11]. For example, Sanfey et al. reported activation of anterior cingulate cortex, anterior insula, and dorsolateral prefrontal cortex when presenting unfair offers vs. fair offers in a single-shot version of the UG [10]. In the single-shot UG, the responder plays just one trial against a single proposer, whereas in the repeated UG, a responder interacts repeatedly with the same proposer. Generally, the behavior in the repeated version of the game is influenced by strategic reasoning and the interaction of the players is more competitive than in the single shot version [12].

However, the statistical analysis used in these studies relies on the comparison of mean blood oxygen level dependent (BOLD) signals calculated from many trials, leaving the question open whether these effects are strong enough to be reliably detected in *single* decisions *before* the decision is revealed by the subject, and without prior knowledge of the actual offer in the trial [13]. Multivariate classification is well suited to such a “brain-reading” task. Brain states have been decoded from the temporal and spatial patterns in fMRI data [14]–[17]. The application of pattern classification to fMRI data was done in the fields of fear perception [18], visual perception [15], goal-related intentions [17], or lie detection [19]. However, in conventional fMRI decoding, these methods are applied offline in the post-experimental analyses. We aimed to predict the decisions before volunteers communicated them and therefore combined the multivariate classification of brain states with real-time fMRI (rtfMRI). This technique allows for online analysis of BOLD activity, for example in the framework of brain computer interfaces [20]–[22]. To date, real-time multivariate analysis of fMRI data has been conducted in very few studies [23]–[25]. La Conte et al. and Sitaram et al. combined whole-brain classification and rtfMRI to implement neurofeedback experiments. Posse et al. combined a classifier with neuroanatomically constrained boosting to analyze rtfMRI data recorded during visual stimulation, finger tapping, auditory attention, and mental calculation. In none of these studies were the online data used to continuously retrain the classifiers during the experiment to improve classification performance.

Here our goal was to discriminate complex brain states occurring in social interactions on the basis of the BOLD signal in a small number of distinct brain regions in real time. Including only few relevant brain areas allowed us to adapt the model parameters of a Relevance Vector Machine (RVM) classifier [26] during the ongoing experiment to improve online classification performance. In a second offline analysis step, we trained a multivariate pattern classifier on the whole brain across subjects and tested the transfer of the brain activation over subjects. This latter step allowed us to *a posteriori* evaluate if the pre-selected brain areas used in the online approach were adequate. We were also able to investigate hypotheses about the role of brain processes related to subjective emotional and motivational states during decision-making and to distinguish them from brain processes related to the evaluation of an objective financial incentive.

## Materials and Methods

### Subjects and paradigm

Ten healthy male subjects (23–28 years, mean: 24.7±1.6 years) with normal or corrected to normal vision were examined after providing written informed consent. The experiments were approved by the local ethics committee of the Medical Faculty of the University of Magdeburg. One subject was excluded from the study after reporting doubts about whether he was playing with human partners. Data from two subjects served for the initial training of the classifier that was subsequently used to examine seven subjects. To avoid cross-gender effects, only male volunteers participated in the study [27].

At the beginning of a session, participants met two male individuals, who were introduced to them as the proposers in the UG. Participants were told that the actual proposer would be chosen randomly from these two individuals for each single trial and that proposers do not interact with each other during the experiment. This procedure was chosen because personal contact between responder and proposer is considered to be an essential prerequisite to establishing a social bond between players [4], [10], [28]. During scanning, the actual offers were made by a computer in a predefined order. This ensured a controllable set of offers.

**Figure 1:**
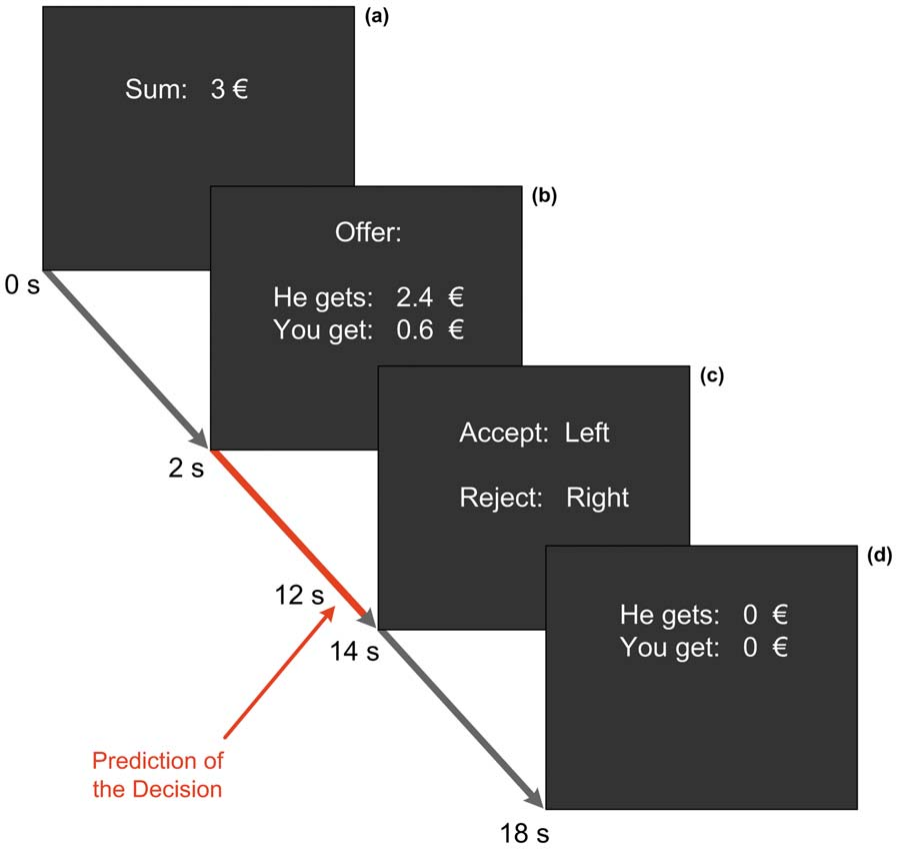
Single trial design in the ultimatum game with cumulative event times. **(a)** Each trial started by displaying the amount to be split (3 euros) for 2 s. **(b)** Subsequently, the offer was shown to the volunteer, who then had 12 s to make up his mind. This time was required for BOLD activity to build up and to subsequently use it to predict the upcoming decision. The classification result was indicated to the experimenter 1–2 s *before* the response screen **(c)** was shown to the participant. During the response phase (4 s), participants pressed one of the buttons to convey their decision. After the response, the payoff (split sum as proposed when the offer was accepted or no money for both players when offer was rejected) in the current trial was presented for 4 s **(d)**. The outcome of a rejected offer is shown.

Brain activity was measured and analyzed using rtfMRI and real-time pattern classification while each volunteer completed 60 trials of 22 s length each. In each trial the amount to be split was shown for 2 s. Subsequently, the offer was shown to the volunteer for 12s. The BOLD signal of the first 10 s after showing the offer was used to predict the upcoming decision. During the following response phase of 4 s length, participants pressed one of the two buttons to convey their decision. Finally, the payoff in the current trial was presented for 4 s and the next trial started immediately (see Fig. 1 for the trial design). The amount of money to share was 3 euros in every trial and five types of offers were presented at the following rates (percentage of 3 euros share for proposer: responder): 6×50∶50, 8×65∶35, 12×70∶30, 21×80∶20, 13×90∶10. These offers were presented in a random order. As usual in economic bargaining games, reimbursement for the volunteers was determined solely by their earnings in the ultimatum game. During the experiment no cumulative earnings were presented. After the experiment, every participant completed a questionnaire to assess whether he had any doubts about having played with a human partner at any time during the experiment. Also the questionnaire assessed the emotional states during the experiment and the perceived decision behavior concerning timing and fairness.

Stimuli were backprojected with an LCD beamer onto a transparent screen. Subjects had to press buttons with their left or right index finger to convey their decisions on the given offers. The mapping between buttons and responses (for either accepting or rejecting) was switched randomly for each trial and displayed at the beginning of each response phase. This prevented the classifiers from using brain activity related to preparation of motor responses [29], [30].

### Imaging protocol and real-time prediction

The blood oxygen level dependent (BOLD) response was measured in a 3 Tesla whole-body MRI scanner equipped with Avanto gradient system (Siemens Medical Systems, Erlangen, Germany). The imaging protocol consisted of a gradient echo EPI sequence for BOLD imaging with repetition time (TR) of 2 s, time to echo (TE) of 29 ms, and a flip angle of 90°. Thirty-one slices with axial slice orientation covering the whole brain were acquired. The matrix size was 64×64 and spatial resolution was 3.4×3.4×4 mm^3^.

**Figure 2:**
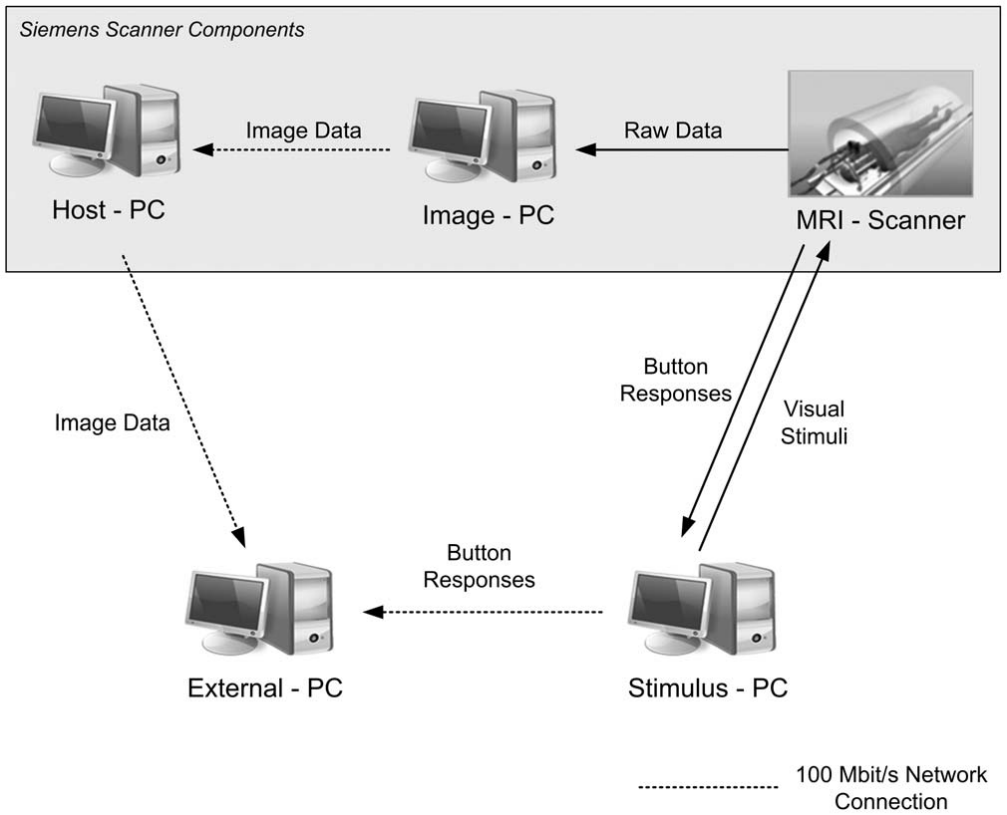
Schema of information flow in the experimental setup. The components highlighted in gray depict the vendor-specific measurement system (Siemens Trio with SYNGO Version VA25A). Initially, the original MR data are fourier-transformed and motion-corrected by the vendor image processing unit (Image PC). The reconstructed data are then transferred to the host computer (External PC). There the data are processed using custom software (rtExplorer). This software performs pre-processing, statistics, online classification, and documentation of the classification results. The participants' responses are processed in the stimulus PC and transferred to the external PC for evaluation of the classification and for retraining the classifier during the ongoing session.

The vendor's EPI BOLD sequence (system version VA25A) and the corresponding image reconstruction programs were modified to export each EPI volume immediately after acquisition and internal motion correction to the host computer of the MR scanner (see Fig. 2 for a scheme of the hardware and the dataflow). All further preprocessing steps, statistical data analysis and classification were performed on an external computer (“External PC” in Figure 2, Pentium IV, 3.0 GHz, 2 GB Random Access Memory, Windows XP) which received the preprocessed EPI volumes via a 100 MBit/s network connection.

**Figure 3:**
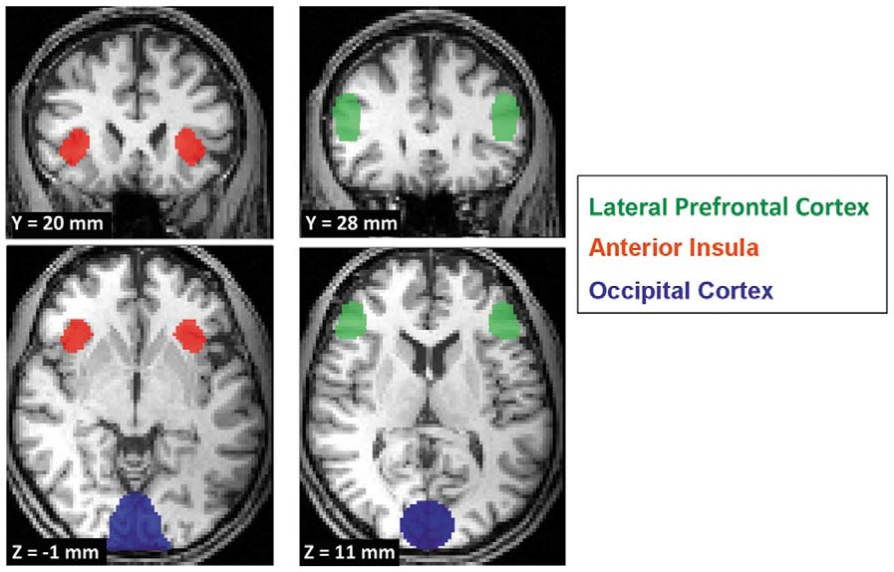
The regions of interest (ROIs) used for online classification projected onto anatomical data of one participant. Three distinct brain regions were used for classifying the volunteers' decisions: anterior insula (AI), lateral prefrontal cortex (LPFC) and occipital cortex (OC). See Table 1 for MNI coordinates and volumes of the ROIs.

The locations of the regions of interest (ROIs) used in the online procedures were pre-specified on the basis of functional MRI data from preliminary experiments including two participants (120 trials) using the same experimental paradigm. The results of a whole-brain offline trained Support Vector Machine (SVM) classifier indicated signal changes predictive of the volunteers' decisions in anterior insula, lateral prefrontal cortex, and occipital cortex (see also Table S1). The informative brain areas revealed in the pilot study were in concordance with those reported in the literature on social interaction where in particular anterior insula and lateral prefrontal cortex were found to be involved in decision making in the ultimatum game [2], [10]. Therefore, we selected prefrontal cortex, anterior insula and visual cortex as ROIs for the online classification. Table 1 lists the MNI coordinates of the centre points and volumes of these ROIs (also shown in Fig. 3).

**Table 1:** Regions of interest used in the real-time classification.

Brain Region	Center Coordinates [mm]			Volume [mm^3^]
	x	y	z	
**LPFC**				
left	−50	28	11	3798
right	50	28	11	3798
**Anterior Insula**				
left	−38.5	20	−1	2925
right	38.5	20	−1	2925
**Occipital Cortex**	0	−88	3	15606

MNI coordinates for the centre points of the regions used for the computation of the t-values. The ROI volume is in mm^3^.

These preliminary data sets were also used to obtain an initial solution for the model parameters of the real-time classifier used in the online experiment. This allowed us to start prediction without first acquiring an exhaustive set of individual data. Importantly, using only a small set of ROIs reduced the feature space sufficiently allowing us to continuously adapt the classifier in real time by retraining with newly arriving individual data.

In the online experiments, custom rtfMRI analysis software was used to process the incoming image data as soon as they were acquired [31]. During online processing, data sets were normalized to 3×3×3 mm^3^ MNI space (Montreal Neurological Institute [32]) and detrended to remove linear signal drifts. The BOLD signal of homologous left and right brain areas were pooled. Then the mean BOLD signal in the ROIs during the baseline period (1^st^ and 2^nd^ scan immediately following the offer) were compared to the mean BOLD signal during the active period (3^rd^ to 5^th^ scan) by calculating one t-value per ROI. Note that we only used data acquired during ten seconds immediately following the presentation of the offer to predict the subject's intended decision in single trials. Thus all data for prediction was acquired before the mapping for the manual decision was revealed. Specifically, we calculated t-values comparing the BOLD response in the first four seconds (scans 1&2) and seconds 6–10 (scans 3&4&5) which were fed into the real-time classification. Because the BOLD response requires approximately five seconds to develop [33], [34] we can use the data acquired in the first four seconds after the offer was presented as a baseline. The BOLD response to the offer can be expected to be fully developed 6–10 seconds after the offer and the difference between BOLD following the offer and baseline is the trial specific effect of the offer.

The three t-values per trial served as input for the online classifier, a nonlinear Relevance Vector Machine Classifier [26] (Software available at www.miketipping.com/index.php?page=rvm), was used to decide on each trial *i* whether an offer would be accepted or rejected. The training set *X* of the classification problem is defined as:
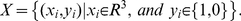
(1)


We refer to *y* as decision vector. Its elements *y_i_* take a value of 1 for an accepted offer and 0 for a rejected offer.

During the experiment, the initial training set (*X_initial_*) was continuously expanded by including the t-values and decision from the *n*-1th trial into the training data (*X_n_*) of the *n*th trial:
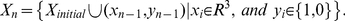
(2)


The classifier was continuously retrained in each trial using the expanded training set. As such, the system adapted the model parameters based on subject-specific activation states in real time and included these in the forecast of volunteers' future decisions to improve classification accuracy.

The RVM applied in online prediction makes use of Bayesian inference to obtain sparse solutions for classification. By computing a posterior distribution, it provides probabilistic classification and has the same functional form as the well-known Support Vector Machines: 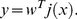
(3)


Here *w* depicts a weight vector and 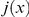 is a kernel function that can be used to express a non-linear relationship between *x* and *y*. The goal is to compute the posterior probability of class membership 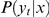 given the input *x* and target class *y_t_*. This is solved by computing the weight posterior 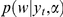, where α denotes a hyperparameter. More details are described in [26].

### Offline estimation of the guessing level of the real-time classifier

To test the reliability of the online prediction, we determined individual empirical guessing levels to ensure that the online discrimination rates were not obtained by pure guessing but exploit information inherent to the data. The theoretical guessing level of a two-class experiment (e.g. accept or reject an offer) is 50% (perfect coin toss). However, other factors, such as the relative frequencies of the two classes in the training set, may influence the classifiers' strategy and bias the guessing level to much higher values than expected [13].

We estimated individual empirical guessing levels by permuting the decision vectors in each subject's data set. Permutation destroys the coherence between the observed BOLD data and volunteers' decisions but retains other information such as class size ratio. The classifier was then retrained, and all trials were classified according to the new training set. These steps were repeated 500 times to estimate the mean guessing level and the 95% confidence interval. Empirical guessing levels were calculated as the geometric mean of the guessing levels for the classes accept and reject [13]. Only if the correct prediction rate of the classifiers in the actual experiment exceeded the 95% confidence interval of the empirical guessing level estimates did we assume that the classifier learned from the inherent structure of the data [35].

### Offline whole brain classification

Additional offline classification was performed to assess classification performance achievable using BOLD data from the whole brain and to further investigate the neural correlates of the decision process. Preprocessing included motion-correction, spatial smoothing with a 9 mm Gaussian kernel, and linear detrending. Furthermore, low-frequency signal fluctuations were removed using a high-pass filter with a cut-off frequency of 0.01 Hz, and BOLD volumes were normalized to 3×3×3 mm^3^ MNI space. Non-brain voxels were excluded by applying a MNI brain template. Before combining the BOLD-data over subjects we first z-scored every subject's data individually. This normalization was done voxel-wise and as a result the BOLD-time series of each voxel had a mean of 0 and a standard deviation of 1. The volumes of the 2^nd^, 3^rd^, and 4^th^ scan after the presentation of the offer were averaged for every subject. This resulted in 420 average functional brain volumes serving as single samples for whole brain classification. Our learning algorithm thus provides a cross-subject model based on single trial data. We then used this to classify the single trial data of the single subject excluded from the classifier training.

The 2^nd^, 3^rd^, and 4^th^ volumes after offer presentation were chosen because the participants reported in the post-scanning questionnaire that they made their internal decisions quickly (i.e. always in less than 5 seconds) after an offer was revealed and always before the accept/reject screen was shown. We thereby also avoided including information about the actual motor response, because in the interval included the participants did not know the mapping of the two buttons for accepting or rejecting the offer.

We used feature selection, a very common approach in pattern classification, to reduce the number of features (voxels) in the input space. This was done on a training set by correlating signal changes with the volunteers' two different decisions. Voxels with correlation values between −0.15 to 0.15 were excluded. Since we wanted to analyze which voxels the trained classifier judged as informative we chose this relatively liberal value to somewhat reduce the number of voxels used for classification without being overly restrictive. Approximately 10^4^ voxels were retained for subsequent classification using this criterion.

For offline classification, we used a publicly available implementation of a SVM [36]. We used a linear classifier because it allows direct analysis of informative features learned during training [37]. Generalization performance was tested in a leave-one-average-volume-out cross-validation (LOOCV) which also included feature selection. In LOOCV, one trial is excluded from feature selection and training. The trained classifier is then used to predict the class label of the excluded trial. These steps are repeated for all trials, and the result (the percentage of correct classified decisions) represents a measure of the generalization power of the classifier. The correct prediction rate is finally calculated as:
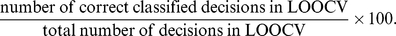



### Guessing level of the whole brain classification and discriminating volume

Theoretical and empirical guessing levels were determined analogous to the approach in real-time prediction, in a permutation test with 500 repetitions.

We extracted the spatial patterns used by the classifier to discriminate between different brain states from the weight vector *w* (Eq. 3). Therefore, *w* was transformed from feature space into the original voxel space and scaled to the length of one. The absolute weight value of each voxel reflects its importance for the discrimination of brain states. To obtain a probability distribution of the weight for each voxel, we permuted the class labels 1000 times. This provides a probability distribution under the null hypothesis of no relationship between class labels and the intrinsic structure of the data [38]. Based on these distributions, we computed the p-values for each voxel to determine which voxels were significantly predictive for the class label. The threshold for the reported discriminating volumes was set to p<0.05 (uncorrected).

## Results

### Behavioral analysis and real-time prediction

**Figure 4:**
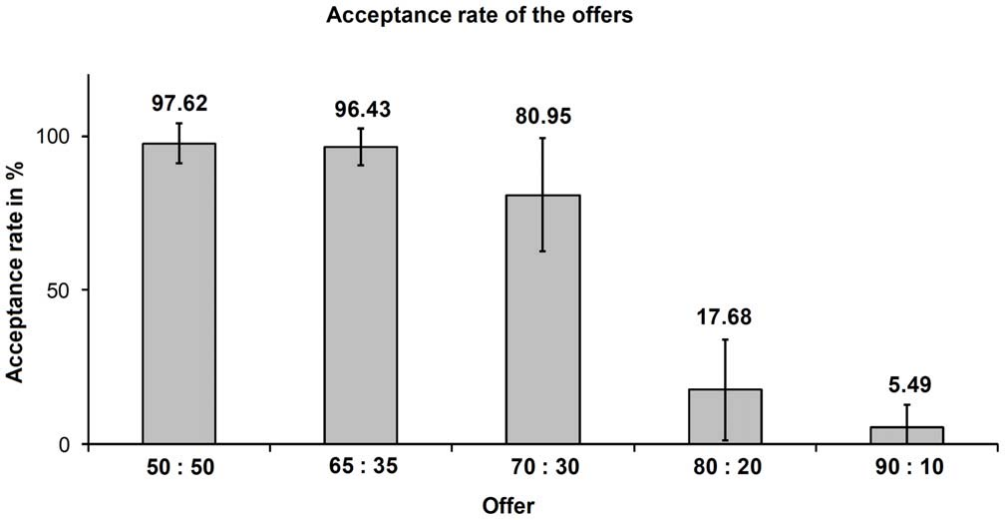
Overall percentage of acceptance rates of the offers in the ultimatum game. Values are calculated as rate of accepted offers over seven volunteers. Labels on the x-axis show the split rate: (proposer: responder).

The percentages of acceptance for the five types of offers are depicted in Figure 4. The acceptance/rejection ratios are in accordance with previous studies employing the repeated UG [39]–[41]. A dramatic drop in the acceptance rate for offers around 20% or less of the amount to be split indicates that these offers were judged as unfair by our participants.

**Figure 5:**
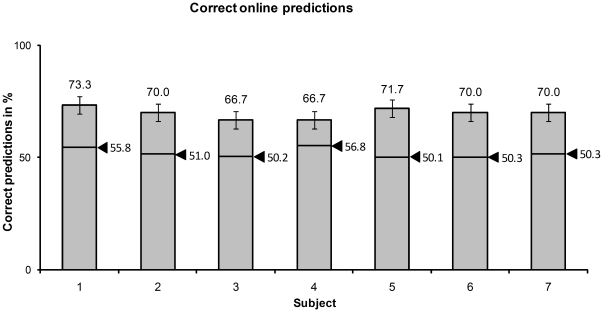
Real-time prediction accuracy of the RVM classifier in the ultimatum game. The arrows mark the empirical guessing levels.

As depicted in Figure 5, the average online prediction accuracy reached 69.7%±2.4%. The average empirical guessing level derived from permutation tests was 52.3%±2.8% (average 2.5% and 97.5% quantiles were 47.2% and 55.3%, respectively). The real-time prediction accuracy was significantly above guessing level (p<0.0038, binomial distribution). The significant prediction results show that the classifier captured information about rejection or acceptance of an offer which was available in brain activity before the participant revealed his decision. With our approach, we were able to predict the participants' decisions 1–2 s before their response (Figure 1). The online processing algorithm (pre-processing, real-time classification) was executed in less than 0.5 s (time required for retraining of the classifier was 0.4 s on average).

**Figure 6:**
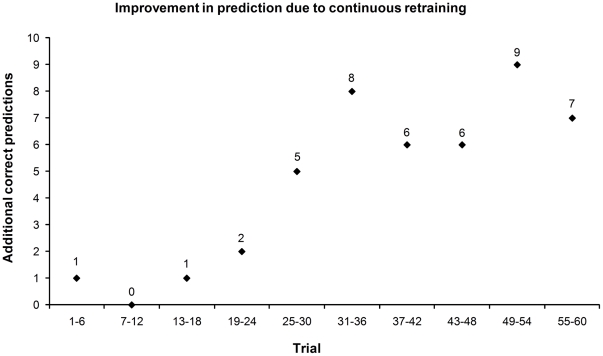
Improvement of online prediction due to continuous retraining. The number of additional correct predictions using individual data acquired during the experiment in a sliding window of six trials are shown. Each window includes 42 single predictions (6 trials times 7 subjects).

To assess the gain in correct predictions achieved by continuously retraining the classifier, we simulated the online procedure both with and without retraining. The overall prediction accuracy increased by 10.7% when novel data were used to retrain the classifier showing a clear benefit of retraining with individual data (Figure 6).

**Figure 7:**
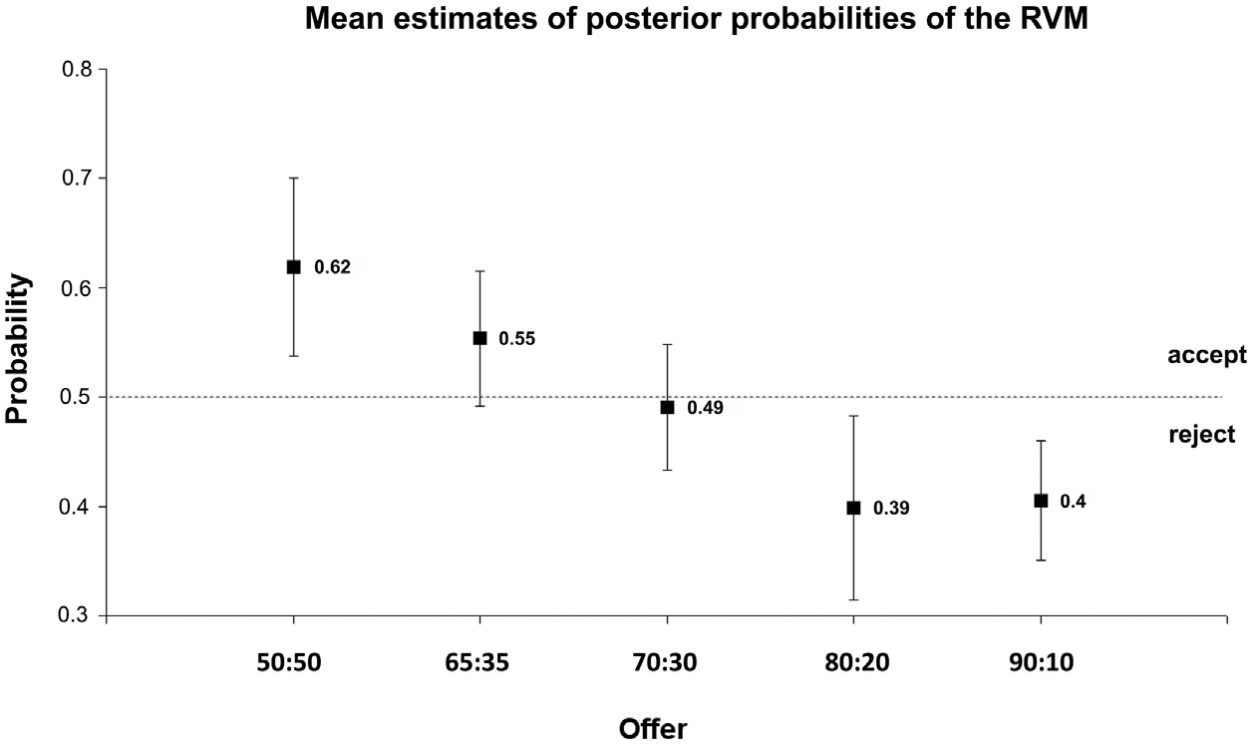
Mean posterior probabilities for accepting an offer assigned by the RVM to single offers in the UG. Means and standard deviations plotted were calculated over the seven volunteers tested in online analysis. The labels on the x-axis depict the split rate: (proposer: responder).

In addition to binary classification accuracy, RVM classification provides a continuous posterior probability estimate for each classified decision. The mean probability estimates for the five types of offers are depicted in Figure 7. Acceptance of an offer is indicated by a probability exceeding 0.5.

**Figure 8:**
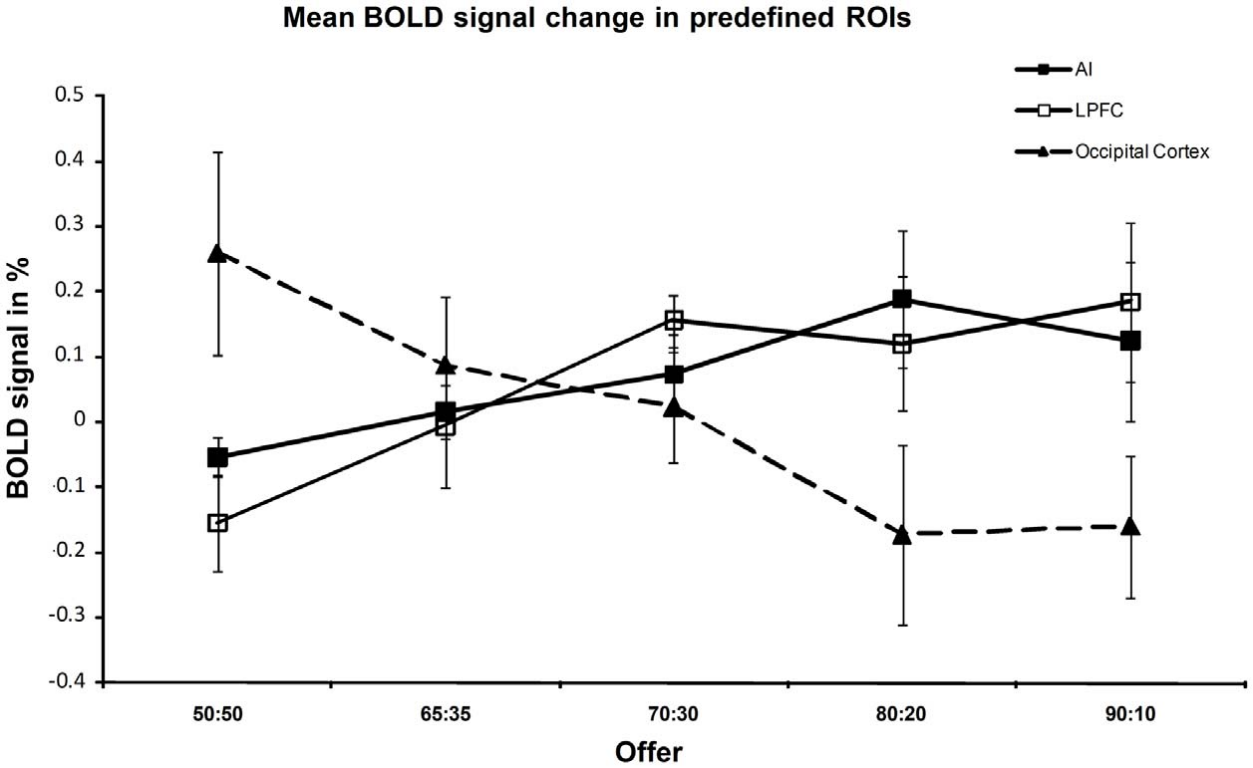
Mean fMRI signal differences in the ROIs used in the online UG to predict acceptance vs. rejection for the five types of offers. Differences were calculated between 1^st^ to 2^nd^ and 3^rd^ to 5^th^ scan after the offer and averaged over the seven participants. Bold signal in AI (slope linear fit 0.062, p<0.05) and LPFC (slope linear fit 0.11, p<0.05). In contrast, signal decreases in OC when the likelihood of acceptance decreases (slope linear fit −0.16, p<0.05).

The analysis of the activation of the signal variation immediately following an offer showed a clear difference between frontal and posterior ROIs. Higher BOLD signal in AI and LPFC predicted rejection, whereas a higher BOLD signal in OC predicted acceptance of an offer (Fig. 8). This finding suggests different functional roles during the evaluation of the offer for frontal and posterior sensory areas.

### Offline whole brain classification

In an additional offline analysis, we pooled the single trial fMRI data from all but one subject (leave on subject out) to train classifiers and test generalization among subjects. This improved the correct classification rate greatly to an average of 81.2%. The average guessing level of the offline classification determined in permutation tests was 51.1%±2.3% SD (average 2.5% and 97.5% quantiles were 47.3% and 55.1%, respectively). Again, the correct classification rate clearly exceeds the 95% confidence interval for guessing. This results clearly shows that there is information about rejection or acceptance of a decision in the BOLD data that is similar among participants. Moreover, this analysis allowed us to derive brain areas informative about a participant's decision from a larger set of subjects and to validate the choice of the ROIs in the online experiment. Table 2 lists the discriminating volumes extracted from the trained linear SVM (see also Fig. S1). Importantly, the brain areas revealed by this analysis include the predefined ROIs used for real-time classification. Both, bilateral LPFC and OC were revealed as informative by the classifier. The only discrepancy was that bilateral AI was used in the online experiment but the offline classifier revealed only right AI as an informative ROI. In addition, offline classification found informative differences consistent over subjects in medial frontal gyrus (MFG), ventromedial prefrontal cortex (vmPFC), ventral striatum (VS), CRUS I in cerebellum, right orbitofrontal cortex (OFC), and posterior superior temporal sulcus (pSTS).

**Table 2:** Volumes discriminative for decisions in the offline classification.

Brain Region	Center Coordinates [mm]			Volume [mm^3^]
	x	y	z	
**Medial Frontal Gyrus**	4	58	6	1629
**Ventromedial PFC**	−2	32	−4	999
**Orbitofrontal Cortex**				
Right	20	58	−8	783
**Anterior Insula**				
Right	36	24	2	1278
**Lateral Prefrontal Cortex**				
Left	−45	28	28	405
Right	40	32	22	2115
**Ventral Striatum**	2	12	−4	1278
**Posterior STS**				
Left	−55	−42	27	1089
Right	54	−46	26	1215
**Occipital Cortex (V1)**	−2	−90	5	4941
**Cerebellum (Crus I)**				
Left	−47	−70	−35	1476
Right	50	−72	−32	1521

MNI coordinates of discriminating volumes found with linear SVM in the offline procedure. Weight values were thresholded at P<0.05 and minimum cluster volume was 300 mm^3^. STS: superior temporal sulcus.

The decision process we investigated so far includes at least two sub-processes: one related to the evaluation of the offer (e.g. low or high earning) and another related to the choice of the response (reject or accept an offer). We analyzed our data according to choices in the previous offline analysis. However, since choice and offer value are correlated over the full scale of offers it is possible that BOLD activity related to evaluation of offer value is more predictive about the subjects' UG responses than choice related BOLD activity, at least on the full scale of offers. To investigate this hypothesis each trial received two labels: one for the offer (low or high) and one for the choice (accepted or rejected) and we trained two classifiers with trials of the same dataset sorted in the two different ways (choice or value). The datasets used for classifier training have to be balanced with respect to each of the four possible label combinations (low/accept, high/accept, low/reject, and high/reject) to avoid unwanted classifier bias. In order to maximize the number of trials available in the four label combinations we distinguish high from low offers around the categorical decision border between 80∶20 and 70∶30 split rates where acceptance rate sharply drops. We labeled 50∶50, 65∶35, and 70∶30 trials as high offers and 80∶20 and 90∶10 trials as low offers. The combination reject/high offer contained the lowest number of samples (n = 19), restricting the number of trials used in the other three combinations in the training of the classifier. In order to avoid selection bias, we evaluated classifier performance on 200 balanced subsets of 76 samples each of which included the 19 rejected/high offers and 19 samples randomly drawn from each of the other three label combinations. The average LOOCV classification accuracy revealed that it was possible to discriminate high from low offers on the basis of the single trial BOLD activity (65.9% correct ±6.2% SD) with some success. On the contrary, discrimination according to choice (accept/reject) was around chance level (56.4% correct ±5.9% SD). This result indicates that brain processes related to the evaluation of offer value rather than the choice related activation allows the prediction of the subject's response on the wide range of offer values used in the offline prediction.

Although no systematic brain activation difference related to choice (reject/accept) may exist over a wide range of offer values, this does not rule out, that a strong link between choice and brain activity exists that may manifest in a predictable and restricted regions along the offer scale where a large change in choices (accept/reject) is found. In the following analysis we aim to demonstrate such an isomorphism between brain activity and behavior for choice related activity. The reasoning behind this analysis follows previous work from us and other groups [42], [43] and is outlined below. We assume that brain activation related to choice should easily discriminate between two adjacent offers if these differ greatly in their acceptance rate and little if they differ little in their acceptance rate. Behaviorally, trials with split rates 50∶50, 65∶35, and 70∶30 trials were mostly accepted and trials with 80∶20 and 90∶10 trials were mostly rejected. Discrimination between trials with different offers within the same category (accepted or rejected) should be low because choice related brain activity should be very similar in trials from the same category. Importantly, choice related brain activity should reproduce the categorical border between acceptance and rejection of offers observed between 70∶30 and 80∶20 split ratios. Consequently, a classifier trained to discriminate between trials either of these two split ratios should produce particularly high discrimination rates because these offers cross the category border between acceptance and rejection. In addition, classifiers trained on adjacent pairs of offers from within a category should be less discriminable.

We tested this prediction by training an SVM in an LOOCV to discriminate between adjacent offers. Therefore, we repeatedly (200 times) selected 42 examples from each split rate. The number of trials used per repetition was limited by the class with the lowest number of examples, in this case the number of trials in the 50∶50 split rate. In concordance with our hypothesis we found the highest discrimination rate between trials from 70∶30 and 80∶20 splits (71.4%±5.53% SD). The single trial discrimination rate was at guessing level for the comparisons among trials between split rates 80∶20 vs. 90∶10 (53.4%±5.3% SD), and 65∶35 vs. 70∶30 (54.6%±4.7% SD), and moderate for the discrimination between split rates 50∶50 vs. 65∶35 (65.9%±5.2% SD). It is important to note that this pattern of results cannot be explained by value differences between offers. The 70∶20 offer differs by 10% (or 0.3 Eurocent) from the 80∶20 offer, the same amount the 80∶20 differs from the 90∶10 and even less than the 65∶35 differs from the 70∶30, and the 50∶50 from the 60∶35 offer (Fig. S3). This result indicates that there exists informative brain activity that reflects choice rather than evaluation of the offer value. The discriminative brain areas found at the choice category border 70∶30 vs. 80∶20 are listed in Table 3 together with those areas discriminative for offers 50∶50 vs. 65∶35 (see also Fig. S2).

**Table 3:** Discriminating volumes found in the offline classification of offers.

Brain Region	Center Coordinates [mm]			Volume [mm^3^]
	x	y	z	
***Classification of offer types 70*** **∶** ***30 vs. 80*** **∶** ***20***				
**Lateral Orbitofrontal Cortex**				
Left	−41	49	−12	1404
Right	38	47	−12	845
**Anterior Insula**				
Right	38	25	1	1836
**Inferior Parietal Sulcus**				
Left	−45	−49	44	459
**Ventral Striatum (N. Accumbens)**	6	14	−10	1080
**Cerebellum (Crus I)**				
Left	−43	−76	−36	702
***Classification of offer types 50*** **∶** ***50 vs. 65*** **∶** ***35***				
**Medial Frontal Gyrus**	3	54	2	3240
**Medial Orbitofrontal Cortex**	−6	28	−12	1485
**Lateral Orbitofrontal Cortex**				
Right	23	55	−12	2160
**Lateral Prefrontal Cortex**				
Right	39	23	30	999
**Inferior Parietal Sulcus**				
Left	−51	−49	42	2214
**Occipital Cortex (V1)**	−4	−93	1	2835
**Cerebellum (Crus I)**				
Left	−43	−73	−35	918

MNI coordinates of discriminating volumes for classification between offers 70∶30 vs. 80∶20 and 50∶50 vs. 65∶35. Weight values were thresholded at p<0.05. Minimum cluster volume was 300 mm^3^.

## Discussion

### Real-time analysis of decision processes

In this study, we show that it is possible to predict the behavior of social agents acting as responders in the UG in real time using BOLD measurements of brain activity to detect complex emotional and cognitive states. Offline analyses confirmed the ROIs selected for online prediction on two pilot subjects and the rejection rates. More detailed analyses of the information about split rate and decision outcome available in the BOLD-data strongly supports the notion that brain activity related to expected subjective value of an offer rather than choice predict the subjects behavior over a large range of offer values. the mere decision process. Importantly, we find that information about choice in the BOLD activity predicts the behaviorally observed categorical change from offer acceptance to rejection.

### BOLD modulation related to emotional and regulatory processes predicts imminent behavior in the UG

We found that AI and LPFC are both predictive of the rejection of an offer on a trial-by-trial basis, in the online as well as in the offline analysis. Both brain areas are involved in emotion regulation and adjustment during social interaction [10], [44]–[48] as well as in the evaluation of negative emotions such as disgust [49], [50]. Increasing activation in AI and LPFC may reflect the experienced level of unfairness which in turn leads to the rejection of the offer in a given trial. In accordance with this interpretation, AI was found to be informative about split level when comparing 70∶30 splits to 80∶20 splits (Table 3) but not when comparing 50∶50 splits to 65∶35 splits. Moreover, this finding is in concordance with Sanfey et al. [10], who also found that higher BOLD activation in AI indicated the rejection of an offer. A competing hypothesis is that activation in AI is not directly connected to the evaluation of negative emotional content but rather refers to attentional processes as reaction to salient environmental stimuli. As part of the ventral attention system the AI is thought to support the reorientation of the attention focus to external stimuli [51]. In this context it was suggested that activation of the ventral attention system may be connected to switching “internally directed” activities to behaviorally salient external stimuli, also in social cognition [52].

As opposed to AI and LPFC, activation in early visual cortex decreased with unfavorable split rates. It has been shown that attention strongly influences the responses of cortical neurons [53], [54]. Different levels of attention elicited by offers with different split rates, i.e. a fair offer may induce stronger attention because it reflects fair behavior and higher monetary outcome, may result in different activation in early visual cortex. However, one could also argue that the behavioral relevance is comparable for high and low offers in the UG and thus should lead to comparable attentional effects. The role of attention-related activation in encoding of decision behavior in the presented social context is not fully explored and may be subject to further investigation.

In sum, the results from the online experiment suggest that activation in brain areas reflecting the subject's emotional and motivational state and self-regulatory processes can be used to discriminate accepted from rejected offers.

### Reward-related brain areas predictive of altruistic punishment and financial incentive

When playing against a computer that is creating offers in a random order, it makes no sense to reject an offer from an economic perspective. Thus, the participants' best strategy to optimize monetary gain would have been to accept any offer. However, responders in our study rejected unfair offers (20% of 3 euros and less) significantly more often than fair offers. This is the behavior expected in the repeated version of the UG (Figure 4) with two humans playing, and corroborates the participants' reports that they thought they were playing with a human. In such a social setting of reciprocal cooperation, altruistic punishment, sacrificing potential monetary gain, can serve to optimize gains in the long run.

Thus, in the ultimatum game the acceptance of an offer is correlated with the expectation of a financial incentive but, in addition, hedonic states following costly punishment of an unfair offer may also contribute to adjustment of behavior [55], [56]. We hypothesized that processing of the financial incentive and altruistic punishment is likely to involve different brain circuits although the same behavioral result, the acceptance or rejection of an offer, is observed [55], [57]. We probed this hypothesis by comparing the discrimination power of brain activity according to financial incentive vs. discrimination power tracking a categorical change from acceptance to rejection signifying altruistic punishment. We found that BOLD activation in VS signified the categorical border and discriminated between offers with a 70∶30 split rate vs. 80∶20 split rate but not between 50∶50 and 65∶35 offers (Table 3 and Fig. S2). The first pair differs with respect to the number of accepted offers, whereas the number of accepted offers is approximately equal and the difference in financial incentive is even higher in the second pair. This implies that, in our social setting, activation in VS, an important component of the reward network, is linked to hedonic states following punishment of unfair offers rather than financial incentive. OFC, another informative brain area of the reward circuit, provides similar information. Interestingly, OFC has previously been linked to the evaluation of threatening and/or punishing stimuli that may lead to the adjustment of behavior [9], [58]. In contrast, ventral medial prefrontal cortices discriminate accepted from rejected offers when all split rates are included (Table 2) but they do not discriminate 70∶30 from 80∶20 split rate trials (Table 3) where the categorical transition between accepted and rejected offers occurred. This suggests that, in contrast to VS and OFC, activation in ventral medial prefrontal cortices is related to the evaluation of monetary gain rather than hedonic states following punishment of unfair offers. This is in agreement with results from previous studies linking ventral medial prefrontal cortices to evaluation of primary as well as secondary rewards like monetary gain [59]. Thus, the result of the offline analysis adds further support to the conclusions that activation in brain areas reflecting the subject's emotional and motivational state and the self-regulatory processes thereof can be used to discriminate accepted from rejected offers in the social UG.

### Cross subject ROI based probabilistic classification

Unlike other offline “mind reading” approaches (compare e.g. [13], [15]), we used a cross-subject approach in the online analysis. Nevertheless, the high prediction rate of 69.7% in the cross-subject procedure confirms the good generalization of the classifier between subjects. This indicates the identification of neural mechanisms that are common between our volunteers. The advantage of this approach is that it allows training of the RVM classifier prior to measurement, simplifying the setup by providing an initial solution of the classification problem without acquisition of additional training trials. Our approach made it possible to predict the subject's choice from the first experimental trial on, although this was with reduced accuracy. Importantly, continuous retraining during the course of the experiment increased classification performance by approximately 11% on average.

Moreover, RVM provides posterior probabilities for single trial class membership, which can be useful in classification-based neurofeedback (compare [23], [25]). Subject-specific offline classification resulted in 81.2% average accuracy and was, as expected, superior to cross-subject online prediction performance. This increase might be partly due to including subject-specific anatomical information but also to the high dimensional feature space we used in offline training. Thus, we would expect improvements in online classification using a more elaborate training scheme that combines non-subject-specific ROI-based classifiers with subject-specific whole-brain classifiers. During an experiment, the classification result would be calculated as a weighted average of the two classification approaches with weights adjusted by the quantity of information available for online classifier retraining. Fast implementations of procedures for preprocessing and training of whole-brain fMRI data are necessary for this approach.

### Implications of single trial online prediction of social decision-making

Whether a responder in the UG finally decides to reject or accept a specific offer depends on a multitude of internal factors. Among these factors are emotions such as the feeling of being treated fairly as well as rational considerations of reward maximization. The extraction of this information about the way a social agent is tending with a decision in real time *before* the decision was actually revealed can have extensive consequences for negotiations and other social interactions. However, the framework presented here for online decision prediction can also be used to study the link between neuronal and behavioral aspects of human decision-making In future studies, this framework could be used to investigate how decision-making processes are influenced by additional information about the emotional or cognitive state of a communication partner in an “augmented communication” scenario which feeds back information about current hidden brain states of the partner. Our approach could significantly extend previous work on effects of overt social cues in social interaction [60], [61], or emotional facial expressions of social agents in bargaining games [62].

### Conclusion

In sum, our results show that, in single trials, it is possible to reliably predict acceptance or rejection of an offer from BOLD measurements of brain activity before the subject reveals the decision with an overt response. However, more detailed analyses indicated that prediction of the decision was based on brain processes related to the perception and evaluation of the offer rather than processes related to the decision itself. Importantly, AI, VS, and LOFC, brain areas related to emotional self-regulation and reward processing for adjustment of behavior, appeared to be strong determinants of overt behavior in the ultimatum game. The decisions derived from the activation in these brain areas paralleled the behaviorally observed categorical transition from high likelihood of acceptance to high likelihood of rejection of an offer when the split rate fell below 70∶30. The framework presented here can be used in future studies to augment information available in social interaction with information about current brain states that remain hidden in traditional approaches.

## Supporting Information

Figure S1 **Discriminating volumes for classification of accepted vs. rejected offers.** The image shows discriminating volumes for the SVM-classification of accepted vs. rejected offers. The threshold is p<0.05 and clusters with a volume lower than 300 mm^3^ were excluded.
(TIF)

Figure S2 **Discriminating volumes for classification of offers 70∶30 vs. 80∶20 and 50∶50 vs. 65∶35.** Shown are the discriminating volumes for the SVM-classification of offers 70∶30 vs. 80∶20 (**A**) and 50∶50 vs. 65∶35 (**B**). The threshold is p<0.05 and clusters with a volume lower than 300 mm^3^ were excluded.
(TIF)

Figure S3 **Regression of prediction accuracies against offer types in balanced set classification.** The figure shows a regression of the correct prediction rate of the balanced classification of each offer against each other offer with the absolute differences of responders earning (in Euro) of the two discriminated offers. For example the rightmost point depicts the classification accuracy in the discrimination of offer 90∶10 vs. 50∶50 (74.58%), which has the maximal difference in earnings for the responder (1.2 Euro).
(TIF)

Table S1 **Discriminating volumes in classification of the pilot study data.** Shown are discriminating volumes of the combined data of two volunteers that participated in a pilot study using the same experimental paradigm as the main study. The results are derived from a multivariate analysis using whole brain classification as described in the methods section of the manuscript in *Offline whole brain classification*. Clusters of a volume lower than 500 mm^3^ are excluded.
(DOC)
