# Supplementary figures and images for: Predicting Decisions in Human Social Interactions Using Real-Time fMRI and Pattern Classification
Source: PLoS One. 2011 Oct 7;6(10):e25304. doi: 10.1371/journal.pone.0025304 (PMC3189203; doi:10.1371/journal.pone.0025304)

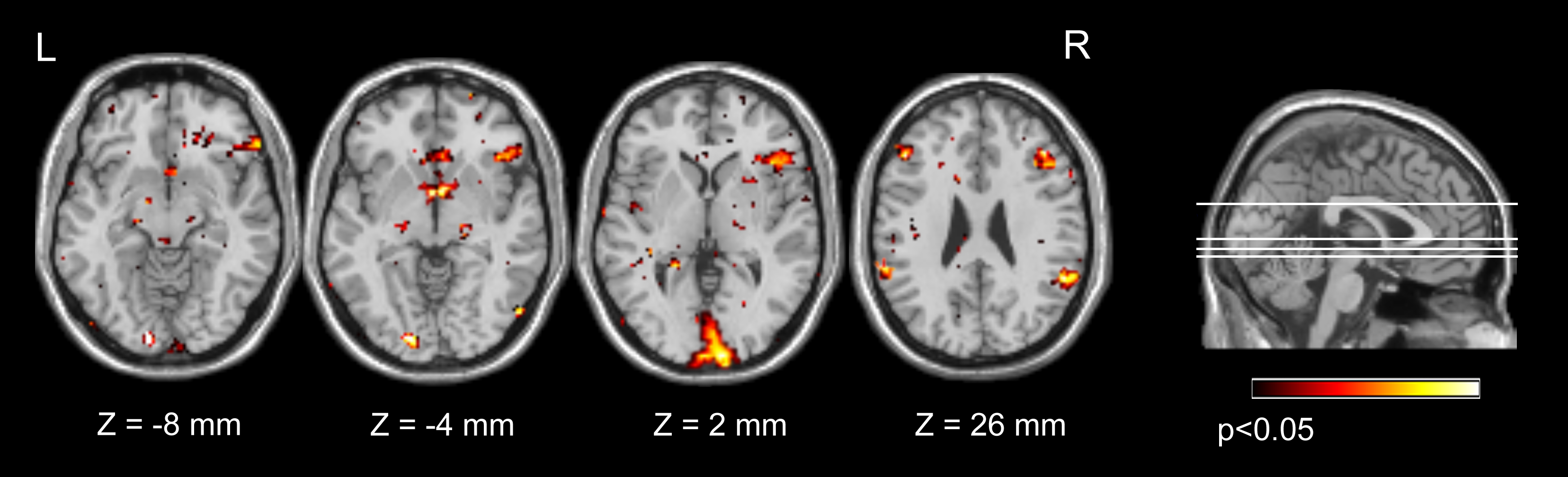

Supplement: Figure S1 — Discriminating volumes for classification of accepted vs. rejected offers. The image shows discriminating volumes for the SVM-classification of accepted vs. rejected offers. The threshold is p<0.05 and clusters with a volume lower than 300 mm3 were excluded. (TIF) [file pone.0025304.s001.tif]

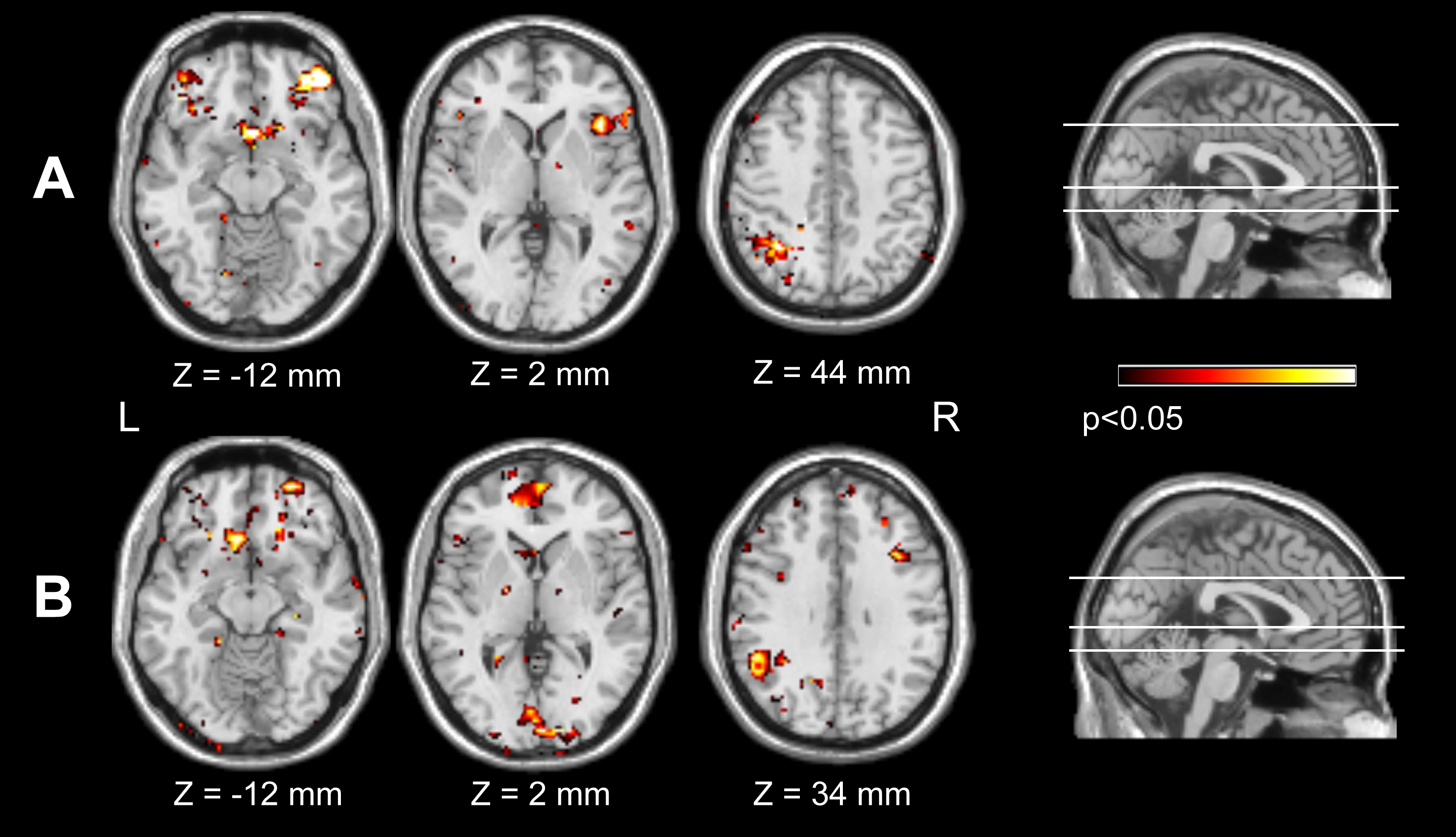

Supplement: Figure S2 — Discriminating volumes for classification of offers 70∶30 vs. 80∶20 and 50∶50 vs. 65∶35. Shown are the discriminating volumes for the SVM-classification of offers 70∶30 vs. 80∶20 (A) and 50∶50 vs. 65∶35 (B). The threshold is p<0.05 and clusters with a volume lower than 300 mm3 were excluded. (TIF) [file pone.0025304.s002.tif]

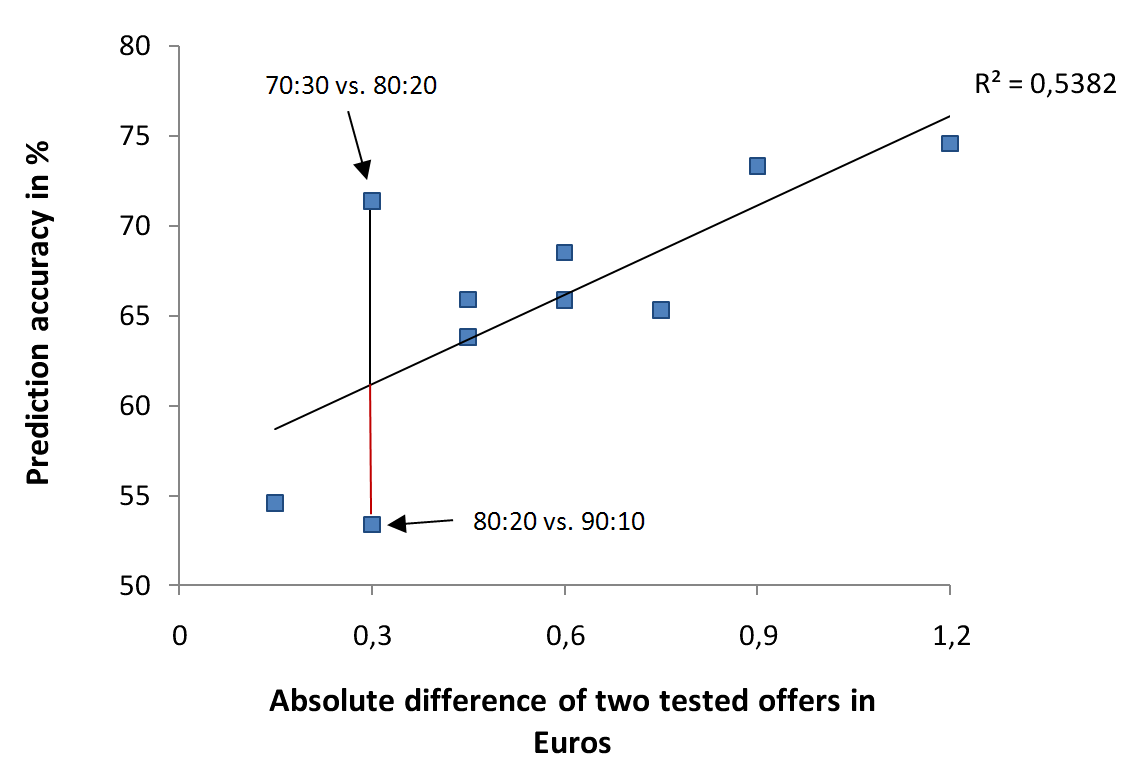

Supplement: Figure S3 — Regression of prediction accuracies against offer types in balanced set classification. The figure shows a regression of the correct prediction rate of the balanced classification of each offer against each other offer with the absolute differences of responders earning (in Euro) of the two discriminated offers. For example the rightmost point depicts the classification accuracy in the discrimination of offer 90∶10 vs. 50∶50 (74.58%), which has the maximal difference in earnings for the responder (1.2 Euro). (TIF) [file pone.0025304.s003.tif]
