# Supplementary material for: Predicting Decisions in Human Social Interactions Using Real-Time fMRI and Pattern Classification
Source: PLoS One. 2011 Oct 7;6(10):e25304. doi: 10.1371/journal.pone.0025304 (PMC3189203; doi:10.1371/journal.pone.0025304)
Supplement: Table S1 — Discriminating volumes in classification of the pilot study data. Shown are discriminating volumes of the combined data of two volunteers that participated in a pilot study using the same experimental paradigm as the main study. The results are derived from a multivariate analysis using whole brain classification as described in the methods section of the manuscript in Offline whole brain classification. Clusters of a volume lower than 500 mm3 are excluded. (DOC) [file pone.0025304.s004.doc]

| Brain Region | Center Coordinates [mm] | | | Volume [mm^3^] |
| --- | --- | --- | --- | --- |
|  | x | y | z |  |
| **Accept vs. Reject** | | | | |
| **Medial Frontal Gyrus** | -2 | 50 | 8 | 1211 |
| **Lateral Prefrontal Cortex** |  |  |  |  |
| Right | 46 | 32 | 13 | 1782 |
| Left | -49 | 29 | 15 | 1306 |
| **Anterior Insula** |  |  |  |  |
| Right | 36 | 24 | 2 | 1278 |
| Left | -45 | 28 | 28 | 732 |
| **Posterior STS** |  |  |  |  |
| Right | 52 | -49 | 21 | 808 |
| **Ventral Striatum** | -1 | 15 | -6 | 704 |
| **Occipital Cortex (V1)** | 3 | -83 | 1 | 2135 |
| **Cerebellum** |  |  |  |  |
| Left | -41 | -68 | -35 | 622 |

**Table S1. Discriminating volumes in classification of the pilot study data.**
